# Transcutaneous Electric Nerve Stimulation Reduces Pathological Sensation of Mesh One Week after Open Inguinal Hernia Surgery: Follow-Up Results from a Randomized, Double Blind and Placebo-Controlled Trial

**DOI:** 10.3390/medicina58060725

**Published:** 2022-05-28

**Authors:** Audrius Parseliunas, Saulius Paskauskas, Violeta Simatoniene, Egle Kubiliute, Edvinas Dainius, Andrejus Subocius, Linas Venclauskas, Donatas Venskutonis

**Affiliations:** 1 Department of General Surgery, Lithuanian University of Health Sciences, 44307 Kaunas, Lithuania; egle.kubiliute@lsmuni.lt (E.K.); edvinas.dainius@lsmuni.lt (E.D.); andrejus.subocius@lsmuni.lt (A.S.); donatas.venskutonis@lsmuni.lt (D.V.); 2 Department of Obstetrics and Gynecology, Lithuanian University of Health Sciences, 44307 Kaunas, Lithuania; saulius.paskauskas@lsmuni.lt; 3 Department of Physics, Mathematics and Biophysics, Lithuanian University of Health Sciences, 44307 Kaunas, Lithuania; violeta.simatoniene@lsmuni.lt; 4 Department of Surgery, Lithuanian University of Health Sciences, 44307 Kaunas, Lithuania; linas.venclauskas@lsmuni.lt

**Keywords:** inguinal hernia, Transcutaneous Electric Nerve Stimulation, quality of life, chronic postoperative pain, mesh sensation

## Abstract

*Background and Objectives*: Quality of life (QoL) and chronic pain are important outcomes following hernia surgery. The long-term effects of Transcutaneous Electric Nerve Stimulation (TENS) on postoperative recovery are not well known. In this trial we investigated the role of TENS on QoL and on the incidence of chronic pain following inguinal hernia repair with mesh. *Materials and Methods*: A total of 80 male patients with elective primary unilateral hernia Lichtenstein repair were randomly allocated to receive TENS or a placebo-TENS procedure. The TENS group received conventional TENS twice a day on the first and second postoperative days. The intensity was set at 0–0.5 mA in the placebo-TENS group. General and hernia-specific QoL, as well as the incidence of chronic pain were assessed using SF-36v2 and the Carolinas comfort scale. *Results*: Less sensation of mesh was reported by the TENS group patients one week after surgery. At this time point, the mean sensation score was 6.07 ± 8.88 in the TENS group and 14.08 ± 16.67 in the placebo-TENS group (*p* = 0.029). Although at two days and one week postoperatively, TENS group patients tended to have less pain, less movement restrictions and better overall hernia-specific QoL, the differences were not statistically significant. At 6 months postoperatively, no incidence of chronic pain was found in either the placebo-TENS or TENS group. *Conclusions*: Conventional TENS applied in the early postoperative period following inguinal hernia repair with mesh was found to reduce mesh-related foreign body sensation one week after surgery. Promising results were also found for other QoL domains.

## 1. Introduction

Inguinal hernia repair is one of the most common surgical procedures performed today. The lifetime occurrence of inguinal hernia is 27–43% in men and 3–6% in women [1]. Open, tensionless repair techniques will be an important treatment for inguinal hernia into the foreseeable future [2]. The Lichtenstein technique is considered to be the reference standard for open inguinal hernia repair. Since the introduction of meshes, recurrence rates have decreased to acceptable and consistent levels of 1–2% [3]. Considerable attention is now directed towards preventing the development of chronic pain, which occurs in 4% to 29% of patients. Chronic pain rates and quality of life (QoL) are the main outcome criteria following hernia repair [4,5,6,7,8,9,10]. Moreover, these outcomes appear to be interrelated [11,12]. A variety of generic [13] and hernia-specific [14,15,16] questionnaires have been used to evaluate QoL following hernia repair.

Pain is the most common complaint reported by patients following inguinal hernia surgery [17]. One of the main risk factors thought to lead to chronic pain development is acute postoperative pain [18]. Postoperative pain has neuropathic, nociceptive and inflammatory origins [12,19,20]. Multimodal access to analgesia using a variety of analgesic medications and techniques, combined with non-pharmacological interventions, is recommended for postoperative pain treatment [21].

Transcutaneous electrical nerve stimulation (TENS) is a noninvasive, inexpensive and easy to learn technique that is used to manage acute and chronic pain of nociceptive and neuropathic origin [22,23]. TENS has a low risk of side-effects and the postoperative analgesic benefit from this treatment has been demonstrated in previous studies following various surgeries. However, the impact of TENS used early in the postoperative period on results from the late postoperative period has yet to be investigated in detail [24,25,26,27,28,29,30,31,32,33,34,35,36,37].

We present here the follow-up results of a randomized, double blind and placebo-controlled trial in which TENS was incorporated into the multimodal treatment of acute postoperative pain following open inguinal hernia repair with mesh. We evaluated the impact of TENS on early and late QoL and on the development of chronic pain.

## 2. Materials and Methods

Details regarding the study design, sample size calculation, randomization and blinding, surgery, postoperative treatment, TENS and placebo-TENS application were described in our previous open access report [38]. Briefly, 80 male patients with unilateral inguinal hernia treated by elective surgery (Lichtenstein technique) were recruited to a prospective, randomized, double blind, placebo-controlled clinical trial. The study was approved by the regional ethics committee (protocol number BE-2-44; 5 June 2018) and registered in the database of clinicaltrials.gov (register number NCT03739060). Eligible patients signed an informed consent form.

Inclusion criteria for the trial were: male gender; primary hernia; no prior TENS procedure; American Society of Anesthesiologists physical status I to III; no cognitive, language, hearing or visual disorders; no movement disorders. Exclusion criteria were: surgical complications; nerves in the inguinal region could not be identified or were damaged; known allergy to a patch glue; chronic use of non-steroidal anti-inflammatory drugs or opioids; neuropathic disease; general contraindication for TENS procedure (such as skin disorder at site of electrode application or pacemaker); non-Lichtenstein modification for hernia repair.

The final sample size was calculated using data on pain ratings when walking at the interim analysis, a two-group design, α = 0.05, and power = 0.80. The sample size for QoL analysis was not calculated separately because this was planned as a secondary outcome of the trial.

**Figure 1:**
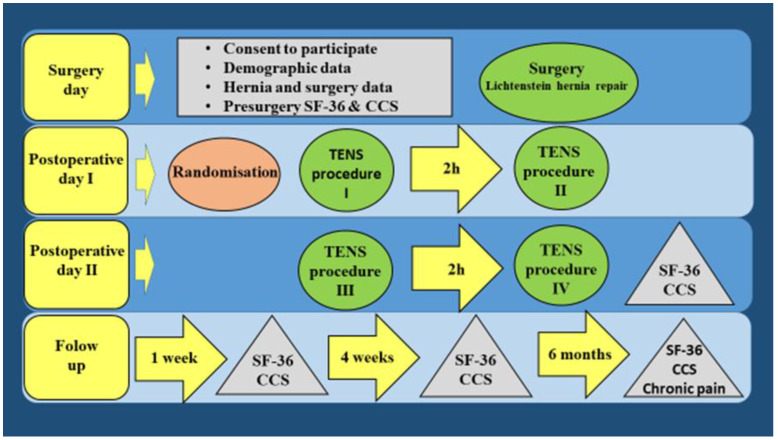
Study design.

On the first postoperative day, participants were randomized to receive active TENS procedures (TENS-group) or inactive TENS procedures (placebo-TENS group) according to a computer-generated random assignment list. Participants and the investigators responsible for outcome measurements and data collection were blinded to the group assignment. Group affiliation was revealed after discharge of the participant and when the investigators could not impact the participant’s future responses. During the first two postoperative days, participants received regular pharmacological analgesia to treat their pain and reach a comfortable level. They could ask for additional doses in case of insufficient analgesia. On the first and second postoperative days, the TENS group received conventional TENS using a constant stimulation frequency of 100 Hz and pulse duration of 200 μs. Segmental and local stimulation was used. Two adhesive electrodes (10 × 5 cm) were placed on the skin at the inguinal region on both sides of the surgical incision. Two adhesive electrodes (5 × 5 cm) were also placed on the skin and parallel to the spine 2 cm from the adjacent spinal processes of the 12th thoracic to 3rd lumbar vertebrae on the surgical side, according to segmental innervation of the inguinal region. The intensity was increased until the participant was able to feel a maximal tolerable tingling sensation without causing discomfort, pain, or muscle contraction. In the placebo-TENS group the whole procedure was the same as in TENS group, but intensity was set at 0–0.5 mA, and participants could not feel any sensations. TENS and placebo-TENS procedures were applied twice a day on the first and second postoperative days, giving a total of 4 procedures for each participant. On the day of discharge from hospital, Ketorolac was prescribed as 10 mg pills and participants were instructed to keep the pain at a comfortable level during daily activities and during rest. The study design is shown in Figure 1.

The outcome measures were general and specific hernia-related QoL in the early and late postoperative period, as well as the incidence of chronic pain and the proportion of symptomatic patients in the TENS and placebo-TENS groups.

General QoL was evaluated using the well-known QoL questionnaire SF-36 v2 short form. This is a reliable and validated measuring instrument that has already been used in the Lithuanian population for different clinical conditions [39,40]. SF-36 includes 8 multiple-item subscales that evaluate physical function, social functioning, role limitations due to physical problems, role limitations caused by emotional problems, mental health, vitality, pain and general health perception. The total score for each SF-36 subscale ranges between 0 and 100, with a higher score indicating better QoL.

Specific (hernia-related) QoL was evaluated using the Lithuanian version of the Carolinas Comfort Scale (CCS) [41]. This has 23 items for the assessment of health-related QoL following hernia repair with mesh. The score for each item is recorded on a Likert-type scale. CCS evaluates QoL during the course of 8 activities: lying down, bending over, sitting up, activities of daily living, coughing or deep breathing, walking, climbing stairs and exercise. The total score is based on a scale of 0–115 and the higher the score, the lower the health-related QoL. CCS scores were calculated according to the CCS scoring algorithm, which allows QoL to be quantified and symptomatic and asymptomatic participants to be identified.

General QoL and specific QoL were assessed before surgery on the admission day, then on the second postoperative day and 1-week, 4-weeks and 6-months postoperatively. At each time point the participants were asked to complete SF-36 and CCS questionnaires. Participants were briefly instructed on how to complete the questionnaires. On the second postoperative day, participants completed the questionnaires before discharge from the hospital. The questionnaires for other time points were mailed to participants, together with stamped, self-addressed envelopes. Participants completed the questionnaires and mailed them back to investigators. This method has been validated against in-person responses or replies obtained by telephone and used successfully in previous trials [4,42,43].

The incidence of chronic pain was determined from the CCS questionnaire completed 6 months after surgery. The criteria for chronic pain were: question b, any activity rated > 2; and question c, any activity rated > 0. Symptomatic patients were identified from the CCS questionnaire completed 6 months after surgery and according to the scoring algorithm. Cases with late postoperative complications were recorded in the follow-up period.

SPSS version 22 software (SPSS, Inc., Chicago, IL, USA) was used for statistical analysis. Categorical variables were analyzed using Pearson’s chi-square test. The Mann–Whitney test for independent samples was used to compare QoL subscales and total scores between the placebo-TENS and TENS groups at different time points. CCS scores were expressed as percentages of the maximum possible score. Pearson’s chi-square test was used to compare the incidence of chronic pain between the groups. Results for all tests were considered significant at *p* < 0.05. Continuous variables were represented as the mean ± Standard Deviation (SD), while categorical variables were represented as percentages.

## 3. Results

**Figure 2:**
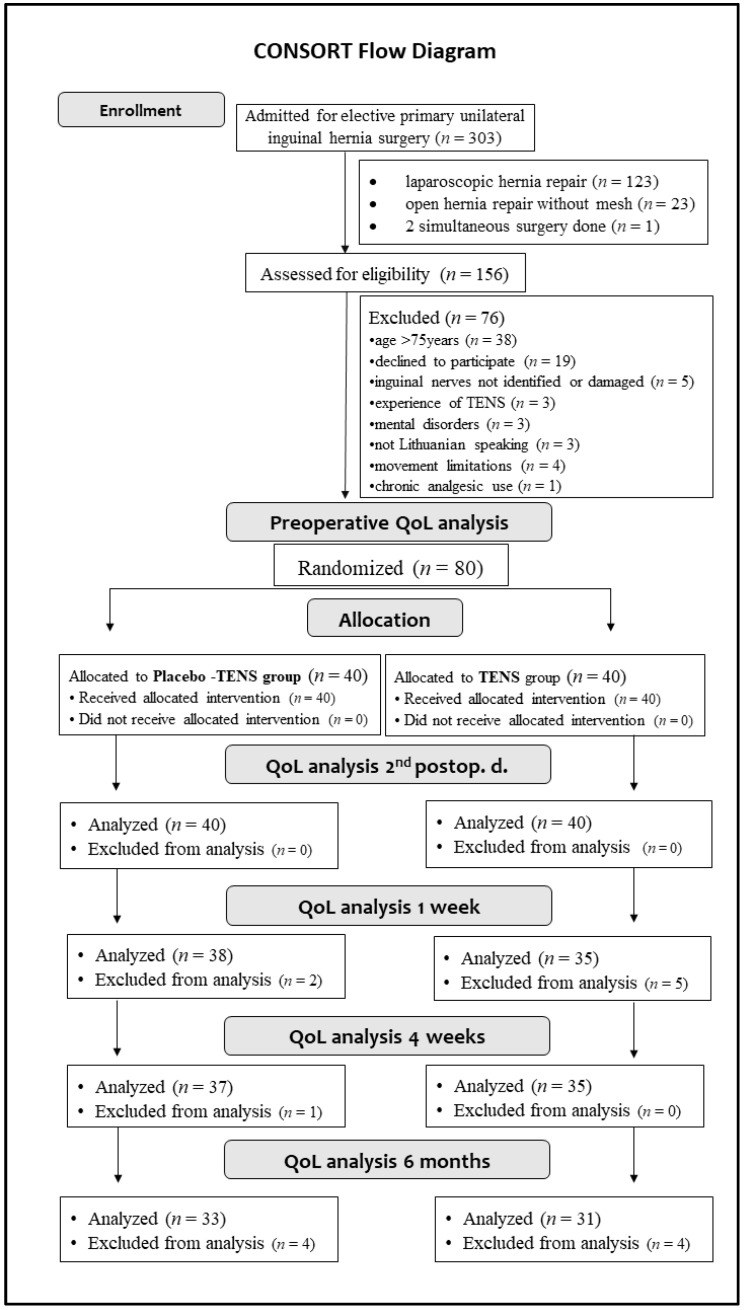
Study flow diagram.

A total of 80 patients were enrolled in the trial and divided equally into the TENS (*n* = 40) group and the placebo-TENS (*n* = 40) group. At the end of the 6-month follow-up period, QoL was analyzed for 33 and 31 participants in the placebo-TENS and TENS groups, respectively. The flow chart for this study is shown in Figure 2. The response rate on the second postoperative day was 100% in both groups. The response rate at the 1-week time point was 95% and 87.5%, at 4 weeks it was 92.5% and 87.5%, and at 6 months it was 82.5% and 77.5%, respectively, for the placebo-TENS and TENS groups.

The TENS and placebo-TENS groups were well matched for all basic characteristics (*p* > 0.05) (Table 1).

**Figure 3:**
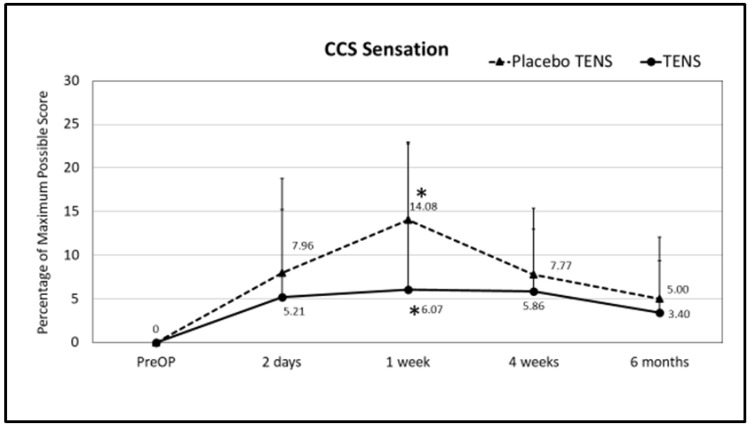
CCS sensation scores. Points represent the percentage of the maximum possible score at different time points expressed as the mean ± SD. Lines represent notional dynamics of the CCS sensation score over time. * Mann-Whitney test, *p* = 0.029.

**Figure 4:**
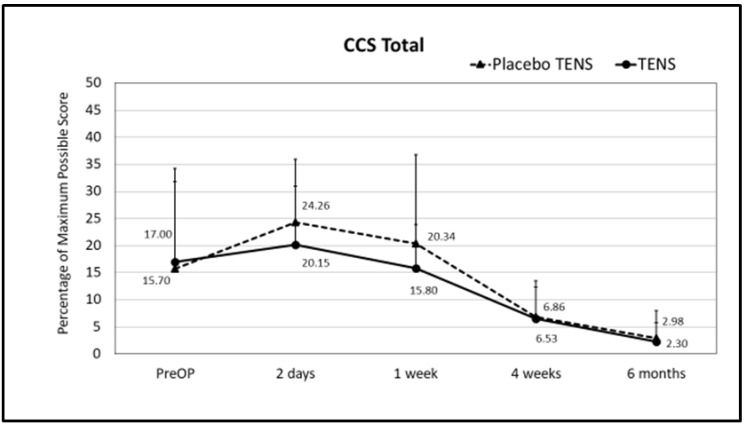
CCS total scores. Points represent the percentage of the maximum possible score at different time points, expressed as the mean ± SD. Lines represent notional dynamics of the CCS total score over time.

**Figure 5:**
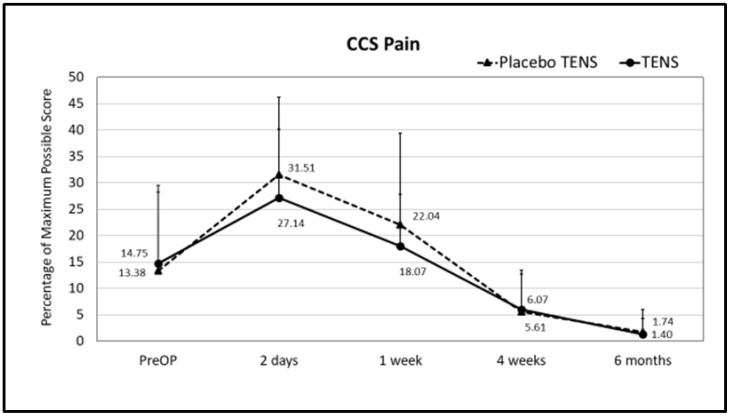
CCS pain scores. Points represent the percentage of the maximum possible score at different time points expressed as the mean ± SD. Lines represent notional dynamics of the CCS pain score over time.

**Figure 6:**
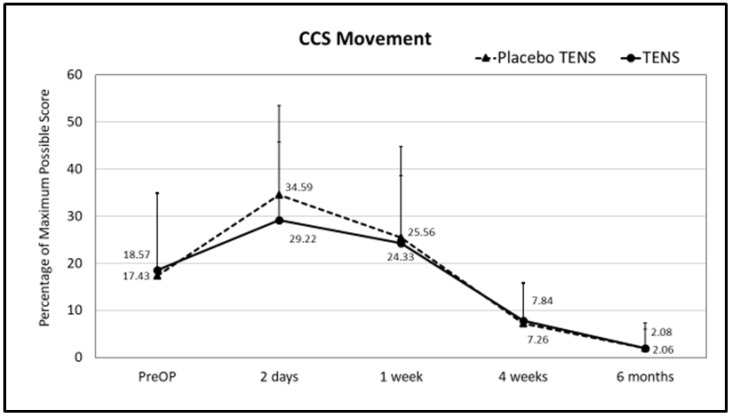
CCS movement scores. Points represent the percentage of the maximum possible score at different time points expressed as the mean ± SD. Lines represent notional dynamics of the CCS movement score over time.

No differences in single domains and in CCS total scores were found between the two groups before surgery. At follow-up, less sensation of mesh was found in the TENS group one week after surgery. The mean sensation score at one week after surgery was 14.08 ± 16.67 in the placebo-TENS group and 6.07 ± 8.88 in the TENS group (*p* = 0.029) (Figure 3 and Figure 4). At the two-days and one-week post-surgical time points, the TENS group tended to have less pain, less restrictions of movement and better overall hernia-specific QoL than the placebo-TENS group (Figure 3, Figure 4, Figure 5 and Figure 6), however the differences were not statistically significant. No differences in the total CCS score and in single domains were found between the two groups at the 4-week and 6-month time points.

**Figure 7:**
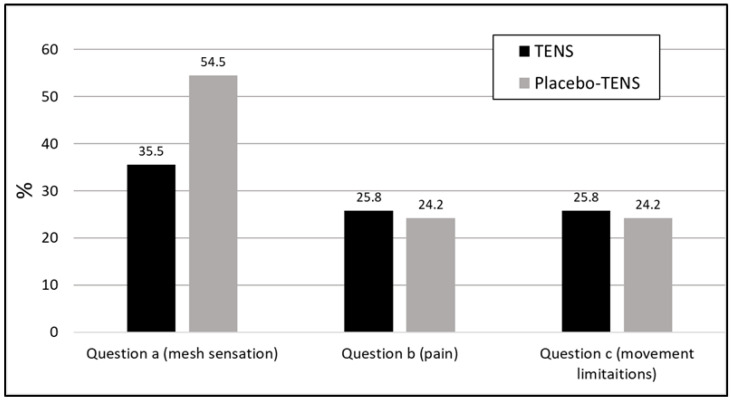
Percentage of responders who scored ≥ 1 at 6 months after surgery.

Fewer responders in the TENS group scored ≥ 1 in the sensation scale compared to the placebo-TENS group (35.5% vs. 54.5%), but the difference was not significant (Figure 7).

**Figure 8:**
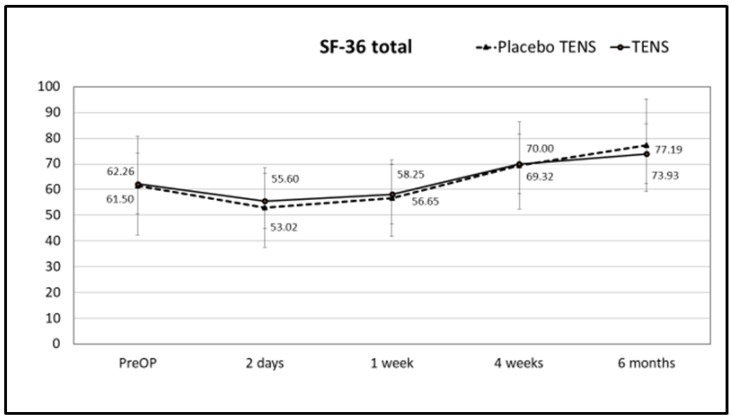
SF-36 total scores. Points represent the score at different time points expressed as the mean ± SD. Lines represent notional dynamics of the score over time.

**Figure 9:**
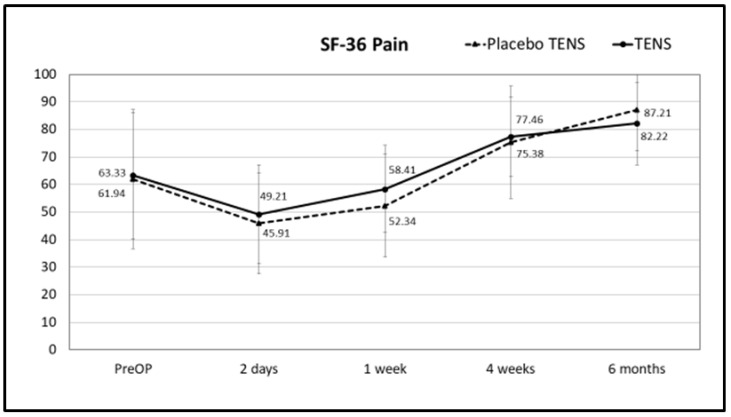
SF-36 pain scores. Points represent the score at different times expressed as the mean ± SD. Lines represent notional dynamics of the score over time.

**Figure 10:**
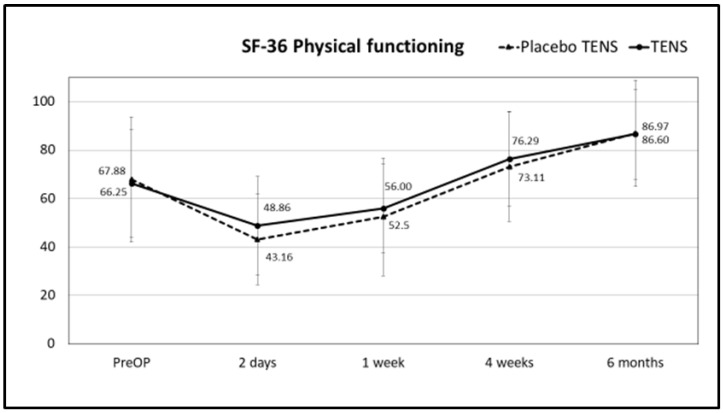
SF-36 physical functioning scores. Points represent the score at different times expressed as the mean ± SD. Lines represent notional dynamics of the score over time.

There were no differences in total SF-36 scores or scores for the different SF-36 domains between the placebo-TENS and TENS groups for general QoL at any of the time points. Results for the SF-36 total score, pain and physical functioning scores are shown in Figure 8, Figure 9 and Figure 10, respectively, while results for the other SF-36 domains are shown in Appendix A Table A1.

No incidence of chronic pain was found in any of the placebo-TENS (0/33) or TENS (0/31) patients at 6-months postoperatively.

A seroma occurred one-week postoperatively in one case from the TENS group and was treated using several punctions. No other surgery or TENS-related complication was registered.

More participants were symptomatic 6 months after surgery in the placebo-TENS group (3/33, 9.1%) than in the TENS group (0/31, 0%), but this did not reach statistical significance (*p* = 0.125).

## 4. Discussion

This prospective, randomized, double blind, placebo-controlled trial found that a relatively short course of TENS integrated into multimodal postoperative pain treatment can reduce the pathological sensation of a foreign body (mesh) one week after open unilateral inguinal hernia repair surgery. However, the impact of an early postoperative TENS course on late general and hernia-specific QoL at different time points remains to be determined, as does the role of TENS in reducing chronic pain following open inguinal hernia repair.

Earlier results from this trial showed that TENS reduces acute postoperative pain and analgesic use after open inguinal hernia repair with mesh [38]. However, it is still not clear whether TENS in the early postoperative period can have positive effects for the late postoperative period. Therefore, in this study we hypothesized that application of TENS during the first two postoperative days following open inguinal hernia repair with mesh has an analgesic effect and also improves the quality of life.

To date, the TENS procedure is known to produce a short-term analgesic effect [45]. TENS has multiple mechanisms of action, including several segmental, extra segmental, peripheral and neurochemical effects [46]. As well as relieving pain, TENS improves peripheral blood flow and accelerates wound healing by acting through the autonomic vascular system [47,48], reduces edema, improves tissue regeneration, reduces the area of tissue necrosis [49] and inhibits inflammation [24,50]. Furthermore, the main effect of TENS is to reduce peripheral and central sensitization associated with pain through various neurochemical mechanisms that include anatomic pathways, neurotransmitters and their receptors [46,51]. Sensitization is defined as increased responsiveness of nociceptive neurons in the nervous system to their normal or subthreshold afferent input. The consequence of central sensitization is that normally innocuous stimuli are perceived as pain, leading to allodynia and hyperalgesia. Persistent acute pain following hernia repair is the main known risk factor for pain chronification [3]. One of the mechanisms for transition from acute to chronic pain is the development and persistence of sensitization [12,52,53,54].

Many patients report a feeling of stiffness or foreign body after implantation of a mesh in the inguinal region following hernia surgery [55]. The pathogenesis of this sensation is not well known, but it is thought the mesh induces a profound inflammatory reaction that leads to a firm scar plate formation with reduced elasticity of the abdominal wall [56]. Reduced-weight and macroporous meshes appear to decrease the immunogenic response and offer the most biocompatibility at the site of implantation [57]. Lightweight meshes are therefore less likely to lead to foreign body sensation than heavyweight meshes [55]. Other risk factors for foreign body sensation have also been considered, such as socio-cultural structure and education [58]. The mechanisms of sensitization may be involved in pathological mesh sensation. In the present study, reduced foreign body sensation was found in the TENS group one week after hernia repair, which might be explained by the anti-inflammatory and anti-hyperalgesic mechanisms of TENS. At two days after surgery, the difference with the placebo-TENS group was smaller and not statistically significant, possibly due to the predominance of pain and limited movement in the first few days after surgery. Hence, the sensation of a foreign body becomes apparent only after the very early period.

In a meta-analysis of open pre-peritoneal versus Lichtenstein repair, chronic postoperative pain of greater than 6 months duration was found in 7.1% and 12.3% of patients, respectively. In another meta-analysis of totally extra peritoneal (TEP) hernia repair versus Lichtenstein, chronic postoperative pain of greater than 3 months duration was reported in 12.5% and 16.8% of patients, respectively [3]. During the follow-up period of our trial, none of the participants in either the TENS or placebo-TENS group had developed chronic pain at 6 months after surgery. This could be due to the strict definition we used for chronic pain, i.e., ≥bothersome moderate pain impacting daily activities and lasting ≥ 3 months postoperatively [3]. It could be argued that pain felt at rest or during daily movements and that does not affect daily activity is not pain. However, even after removing the activity restriction criterion, no cases of chronic pain were found. Unfortunately, there is no single definition of chronic postoperative pain and hence it is difficult to compare and interpret the results of different trials [59]. In our opinion, a uniform method for the evaluation of chronic pain is required. CCS could provide a sensitive, objective and standardized tool to identify patients with chronic pain.

**Figure 11:**
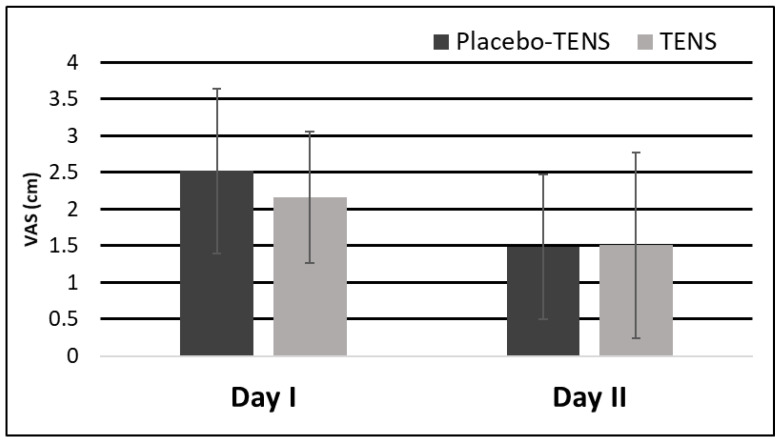
Mean VAS pain (cm) during walking on the first and second postoperative days before the TENS procedure.

Another reason for the development of chronic pain is the presence and poor control of risk factors [3], such as insufficient control of acute postoperative pain. It is also important to note that all participants in the current trial were males who underwent surgery for primary inguinal hernia, with the surgery performed by experienced surgeons. Postoperative pain was well controlled during the first two postoperative days, regardless of the group to which the subject was assigned (Figure 11). However, the absolute and relative pain relief was higher in the TENS group compared to the placebo-TENS group [38]. If needed, patients in both groups controlled pain with additional doses of analgesics. Therefore, we suggest that pain control at home or in the outpatient setting could be worse if patients are discharged from hospital on the day of their surgery. We found that participants in the TENS group used fewer analgesics to control acute postoperative pain [38]. Considering simplicity and safety use of TENS, it could be administered by the patients themselves to control acute postoperative pain.

We believe that good postoperative pain control and the absence of other risk factors can reduce the incidence of chronic pain.

We also evaluated the impact of TENS on specific hernia-related QoL following open hernia surgery with mesh. Total CCS scores and results for other domains at the two-day and 1-week postoperative time points were promising. The TENS group had a lower total CCS score than the placebo-TENS group, but this did not reach statistical significance. After one month and 6 months postoperatively, no differences were apparent between the two groups for all CCS domains and for the CCS total score. At 6-months postoperatively, fewer patients in the TENS group tended to respond positively to the mesh perception question compared to the placebo-TENS group, with less symptomatic patients identified in the TENS group. A statistically significant difference was not apparent between the two groups. The main reason for this was likely to be the smaller number of patients analyzed at these time points. Indeed, this was a major limitation of the follow-up part of this trial.

Another reason why TENS did not impact the long term QoL may have been the short course of TENS therapy. Extending the course is likely to result in a greater benefit from TENS. Observational studies on the efficacy of long-term TENS for the treatment of chronic pain from various causes have shown that it reduces pain interference with work, home and social activities. Long-term TENS also increased activity levels and pain management, decreased the use of other therapies (e.g., physical therapy, occupational therapy), and decreased the use of narcotics, tranquilizers, muscle relaxants, nonsteroidal anti-inflammatory drugs and steroids [60,61]. In more recent studies, the duration of TENS therapy has exceeded 6 months. A similar duration of treatment could be used to treat chronic pain that develops after inguinal hernia surgery. It is not known what the duration of TENS therapy should be for the treatment of acute postoperative pain in order to also obtain a long-term benefit from pain relief.

The main limitation of the study was that sample size calculation was based on acute pain outcomes in specific on pain ratings when walking. Therefore, secondary outcome analysis was not enough powered. Perhaps this is the reason why the difference in general and specific hernia-related quality of life outcomes were not found. Other limitations of this study were the selection of participants according to narrow criteria (male gender, primary hernia on one side) and a single-center study site.

In conclusion, conventional TENS applied in the postoperative period following inguinal hernia repair with mesh has an analgesic effect. It also reduces mesh-related foreign body sensation one week after surgery and shows promising results for other QoL domains. We suggest that physicians involved in hernia surgery should consider integrating conventional TENS into multimodal postoperative pain treatment. Additional good quality trials with a larger sample size are needed to confirm the potential benefit of TENS for reducing the incidence of chronic pain and for improving late postoperative QoL following hernia repair.

## 5. Conclusions

Conventional TENS applied in the postoperative period following inguinal hernia re-pair with mesh has not only analgesic effect but also reduces mesh-related foreign body sensation one week after surgery.

## Figures and Tables

**Table 1:** Basic characteristics of the trial participants.

Characteristics	Placebo-TENS Group	TENS Group	*p*-Value
Age, years (mean ± SD)	61.08 ± 12.51	61.77 ± 10.84	0.793
BMI, kg (mean ± SD)	26.10 ± 2.99	26.75 ± 3.78	0.398
ASA Physical status (%)			
I	32.5	22.5	
II	57.5	52.5	
III	10.0	25.0	0.184
Inguinal hernia side (%)			
Right	57.5	65.0	
Left	42.5	35.0	0.491
Inguinal hernia type (%)			
lateral	70.0	67.5	
medial	30.0	32.5	0.809
Inguinal hernia size EHS * (%)			
1	5.0	2.5	
2	47.5	47.5	
3	47.5	50.0	0.836
Surgery duration, min. (mean ± SD)	75.25 ± 18.95	76.38 ± 18.40	0.788
Preoperative CCS score (mean ± SD):			
Total	15.70 ± 18.51	17.00 ± 14.80	0.324
Pain Score	13.38 ± 16.15	14.75 ± 13.48	0.377
Movement Score	17.43 ± 21.10	18.57 ± 16.47	0.307
Preoperative SF-36 total score (mean ± SD)	61.50 ± 19.07	62.26 ± 11.80	0.866

* hernia size according to European Hernia Society (EHS) classification [44].

## Data Availability

The data presented in this study are available on request from the corresponding author.

## Appendix A

**Table A1:** SF-36 domains at different time points.

Time Point	SF-36 Domain	TENS Group	Placebo-TENS Group	*p*
Surgery day	Total	62.26 ± 11.80	61.50 ± 19.07	0.866
	Physical Functioning	66.25 ± 22.35	97.88 ± 25.79	0.609
	Role-Physical	52.97 ± 17.90	51.09 ± 26.28	0.482
	Bodily Pain	63.33 ± 22.95	61.94 ± 25.27	0.868
	General Health	47.88 ± 13.77	51.75 ± 16.19	0.305
	Vitality	64.06 ± 15.49	65.47 ± 19.81	0.677
	Social Functioning	70.31 ± 19.95	68.44 ± 23.85	0.814
	Role-Emotional	62.92 ± 18.87	57.92 ± 25.73	0.282
	Mental Health	70.38 ± 16.89	67.50 ± 19.90	0.605
2 days	Total	55.60 ± 10.67	53.02 ± 15.34	0.635
	Physical Functioning	48.86 ± 20.30	43.16 ± 18.65	0.329
	Role-Physical	41.25 ± 20.18	37.66 ± 22.35	0.709
	Bodily Pain	49.21 ± 17.93	45.91 ± 18.30	0.430
	General Health	50.86 ± 14.68	53.16 ± 15.09	0.523
	Vitality	65.36 ± 15.03	64.31 ± 19.27	0.996
	Social Functioning	61.43 ± 19.97	58.22 ± 25.88	0.587
	Role-Emotional	53.10 ± 19.40	52.63 ± 25.93	0.826
	Mental Health	74.71 ± 16.62	69.08 ± 22.63	0.399
1 week	Total	58.25 ± 11.38	56.65 ± 14.81	0.956
	Physical Functioning	56.00 ± 18.50	52.50 ± 24.29	0.706
	Role-Physical	38.93 ± 19.42	40.46 ± 21.14	0.530
	Bodily Pain	58.41 ± 15.79	52.34 ± 18.61	0.394
	General Health	55.00 ± 14.19	56.32 ± 16.67	0.660
	Vitality	66.79 ± 16.17	66.45 ± 17.40	0.960
	Social Functioning	63.21 ± 17.92	61.18 ± 24.79	0.690
	Role-Emotional	93.81 ± 19.53	51.97 ± 24.54	0.733
	Mental Health	73.86 ± 15.10	71.97 ± 19.37	0.846
2 weeks	Total	70.00 ± 13.10	69.32 ± 16.94	0.830
	Physical Functioning	76.29 ± 19.64	73.11 ± 22.62	0.575
	Role-Physical	56.61 ± 16.32	57.26 ± 24.94	0.931
	Bodily Pain	77.46 ± 14.38	75.38 ± 20.31	0.872
	General Health	57.00 ± 17.95	59.32 ± 15.91	0.614
	Vitality	71.07 ± 14.86	71.96 ± 17.16	0.517
	Social Functioning	78.57 ± 17.83	74.66 ± 20.93	0.450
	Role-Emotional	68.57 ± 19.81	66.89 ± 26.97	0.936
	Mental Health	74.43 ± 18.58	75.95 ± 17.83	0.730
6 months	Total	73.93 ± 12.43	77.19 ± 18.06	0.206
	Physical Functioning	86.60 ± 18.52	86.97 ± 21.86	0.569
	Role-Physical	73.00 ± 20.86	74.62 ± 26.33	0.543
	Bodily Pain	82.22 ± 15.04	87.21 ± 14.99	0.199
	General Health	54.80 ± 18.40	58.33 ± 19.54	0.463
	Vitality	70.00 ± 16.24	74.81 ± 19.79	0.226
	Social Functioning	79.50 ± 16.88	83.33 ± 21.35	0.191
	Role-Emotional	73.33 ± 20.97	75.25 ± 28.07	0.486
	Mental Health	72.00 ± 13.69	76.97 ± 19.20	0.196
